# A new homogeneity index definition for evaluation of radiotherapy plans

**DOI:** 10.1002/acm2.12739

**Published:** 2019-10-12

**Authors:** Lingling Yan, Yingjie Xu, Xinyuan Chen, Xin Xie, Bin Liang, Jianrong Dai

**Affiliations:** ^1^ Department of Radiation Oncology National Cancer Center/National Clinical Research Center for Cancer/Cancer Hospital Chinese Academy of Medical Sciences and Peking Union Medical College Beijing China

**Keywords:** new homogeneity index, percentage accuracy, radiotherapy plan evaluation

## Abstract

**Purpose:**

The goal of this study was to define a new homogeneity index (HI) to evaluate dose homogeneity within a target volume.

**Materials and Methods:**

The new HI is based on the area under an ideal dose‐volume histogram curve (IA), the area under the achieved dose‐volume histogram curve (AA), and the overlapping area between the IA and AA (OA). It is defined as the ratio of the square of OA to the product of the IA and AA. To evaluate the performance of the new HI, 88 cases were selected and two plans were designed for each case. The homogeneity of the two plans was first evaluated by three physicists, with their judgments forming the evaluation standard and then evaluated by the new HI and other HIs of *D*_max_/*D_p_*, *D*_5_/*D*_95_, (*D*_2_ − *D*_98_)/*D_p_*, (*D*_2_ − *D*_98_)/*D*_50_ and S‐index. An evaluation was determined to be accurate if its result was agreed upon by physicists. The percentage accuracy of evaluation was calculated as the ratio of the number of accurate evaluations to the total number of evaluations. Pearson's chi‐square test was performed for statistical analysis.

**Results:**

The percentage accuracies of the new HI, *D*_max_/*D*_p_, *D*_5_/*D*_95_, (*D*_2_ − *D*_98_)/*D_p_*, (*D*_2_ − *D*_98_)/*D*_50_, and S‐index were 98.51%, 88.80%, 94.78%, 94.78%, 96.27%, and 97.01%, respectively. The newly defined HI had the highest accuracy of all the HIs, with the difference being statistically significant (*P* < 0.05).

**Conclusions:**

The newly defined HI was shown to be effective in the evaluation of dose homogeneity, and we recommended it for evaluating the homogeneity of radiotherapy plans.

## 1 INTRODUCTION

The delivery objective of irradiation therapy is to homogeneously deliver 100% of the prescribed dose to the target volume. Unfortunately, external‐beam radiotherapy involves a balance between the prescribed dose delivered to the planning treatment volume and the healthy tissue tolerance.^1^, ^2^ Although intensity modulation technology is capable of delivering superior dose distributions tailored to the geometry of the structures to be irradiated, it often produces inhomogeneous dose distributions.^3^, ^4^, ^5^ It is therefore necessary to evaluate the homogeneity of radiotherapy plans before performing radiotherapy.^6^, ^7^, ^8^

Homogeneity index (HI) is a simple and fast scoring tool for analyzing and quantifying dose homogeneity in the target volume. It can be used to compare the dose distributions among different radiotherapy plans, so that better quality plans can be available. It can also compare various devices or techniques and serve as a guide to develop the future technology and treatment protocols. In turn, this can help us to find the means by which treatment plans can be improved in future.

Several HIs have been reported in the literature,^9^, ^10^, ^11^, ^12^, ^13^ including *D*_max_/*D_p_*, *D*_5_/*D*_95_, (D_2_ − *D*_98_)/*D_p_*, (*D*_2_ − *D*_98_)/*D*_50_, and S‐index. The conventional HI of *D*_max_/*D_p_* is defined as the ratio of the maximum dose (*D*_max_) in the target volume to the prescribed dose (*D_p_*), with a value closer to one indicating better homogeneity.^9^ The *D*_max_ value is sensitive to calculation parameters, such as grid size and grid placement, so the *D*_max_/*D_p_* index may not be reliable. The HI of *D*_5_/*D*_95_, choosing the minimum dose in a target volume rather than a dose point, is described as the ratio of the minimum dose in 5% of the target volume (*D*_5_) to the minimum dose in 95% of the target volume (*D*_95_).^10^ Another HI is calculated as (*D*_2_ − *D*_98_)/*D_p_*,^11^ where *D*_2_ and *D*_98_ are the minimum dose covering 2% and 98% of the target volume respectively, although report 83 of the ICRU suggests (*D*_2_ − *D*_98_)/*D*_50_ instead,^12^ with *D*_50_ being the normalization value. Lower values of (*D*_2_ − *D*_98_)/*D_p_* and (*D*_2_ − *D*_98_)/*D*_50_ indicate a more homogeneous dose distribution. It should be noted that the HIs of *D*_max_/*D_p_*, *D*_5_/*D*_95_, (*D*_2_ − *D*_98_)/*D_p_*, and (*D*_2_ − *D*_98_)/*D*_50_ are usually based on two or three points of the dose volume histogram (DVH) curve, and do not reflect information from the whole DVH. Differing from the above mentioned HIs, the S‐index proposed by Yoon et al.^13^ takes the whole DVH into consideration, using the standard deviation of the differential DVH curve to quantify the dispersion of the average dose of the target volume. While the information contained in the S‐index is relatively unitary, the S‐index only reflects information from the achieved dose‐volume histogram curve (A‐DVH); it does not make reference to any information from the prescribed dose.^14^

An ideal HI for evaluating radiotherapy plans should objectively and accurately reflect the dose distribution. We therefore developed a new HI to evaluate the dose homogeneity of the radiotherapy plans, incorporating information from the ideal dose‐volume histogram (I‐DVH) curve and the A‐DVH curve, and we demonstrate its application to two clinical examples. In addition, we evaluated the percentage accuracy of the new HI and *D*_max_/*D_p_*, *D*_5_/*D*_95_, (*D*_2_ − *D*_98_)/*D_p_*, (*D*_2_ − *D*_98_)/*D*_50_ and S‐index.

## 2 MATERIALS AND METHOD

### 2.1 Definition of the new HI

**Figure 1:**
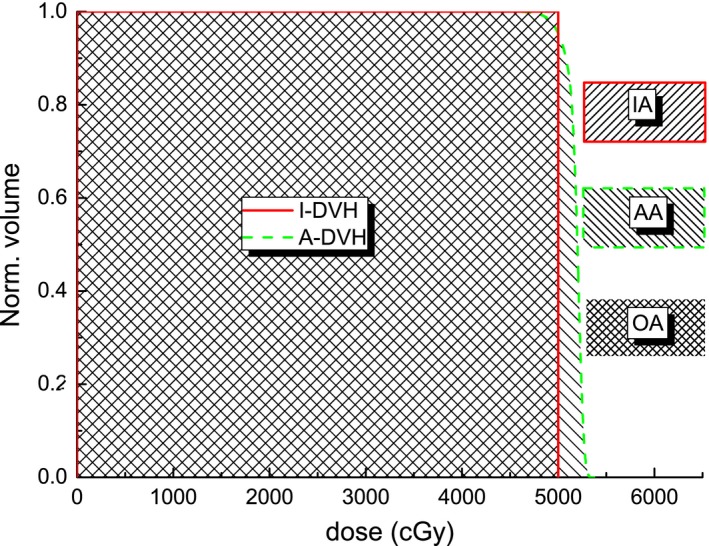
DVHs of example radiotherapy plan. Solid line represents I‐DVH; Dash line represents A‐DVH. A‐DVH, achieved dose‐volume histogram curve; DVH, dose volume histogram; I‐DVH, ideal dose‐volume histogram.

The ideal DVH for a target volume would be a step function, with 100% of the target receiving exactly the prescribed dose. However, the A‐DVH deviates from this step function. The new HI can be defined as:

(1) $$\text{HI}=\frac{{\text{OA}}^{2}}{\text{IA}*\text{AA}}$$

where IA is the area under the I‐DVH curve, AA is the area under the A‐DVH curve, and OA is the overlapping area between IA and AA. The relationship between IA, AA, and OA is shown in Fig. 1 for an example radiotherapy plan with a steep dose gradient. When the I‐DVH and A‐DVH are completely overlapping and the dose distribution inside the target volume is homogeneous, the new HI has a value equal to 1, and so the closer the new HI is to a value of 1, the more homogeneous is the dose.

### 2.2 Construction of the I‐DVH

**Figure 2:**
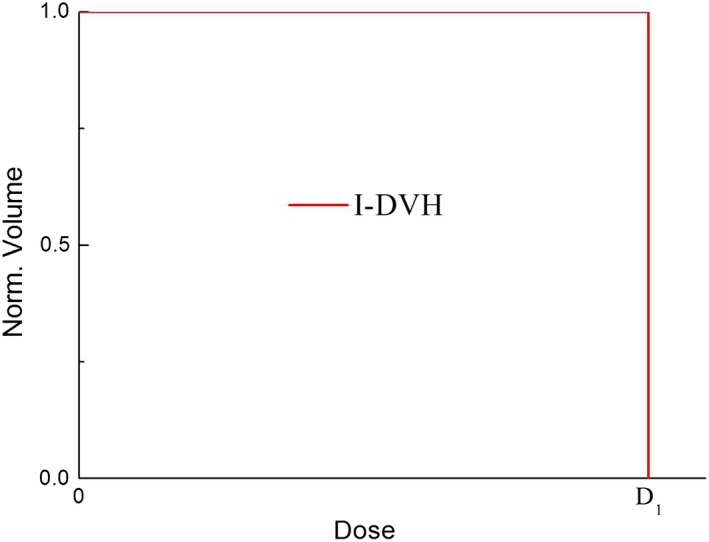
I‐DVH of PTV_1_ for example radiotherapy plan with one steep dose gradient. I‐DVH, ideal dose‐volume histogram; PTV, planning target volume.

**Figure 3:**
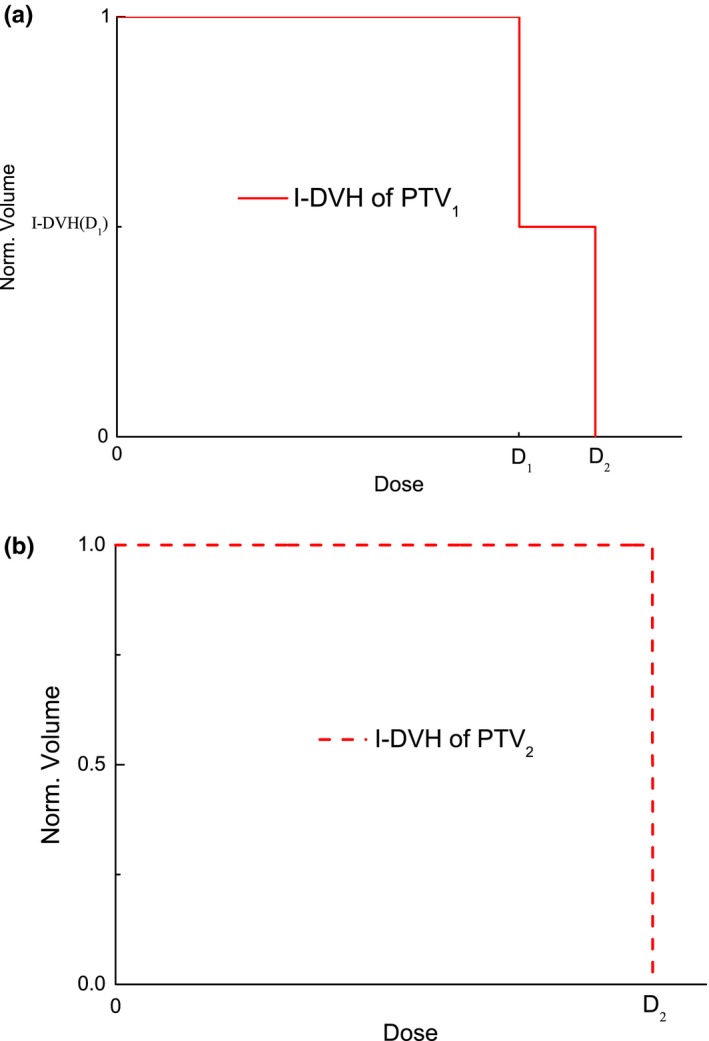
I‐DVH of PTV_1_ (a) and PTV_2_ (b) for example radiotherapy plan with two steep dose gradients and two target volumes. I‐DVH, ideal dose‐volume histogram; PTV, planning target volume.

Hypothesis: there are M different prescribed doses, defined as *D_i_* (*i* = 1, 2, …, M), and $D_{1}<D_{2}<D_{3}\cdots <D_{M}$. For each prescribed dose, there are *N*_i_ target volumes. The I‐DVH of a target with prescribed dose *D*_i_ is defined as follows:

(2) $$I-\text{DVH}\left(D_{i}\right)=V\left[\overset{N_{i}}{\underset{1}{⋃}}\left({\text{PTV}}_{i}\right)⊗\overset{M}{\underset{j=i+1}{⋃}}\overset{N_{j}}{\underset{1}{⋃}}\left({\text{PTV}}_{j}\right)\right]/V\left[\overset{N_{i}}{\underset{1}{⋃}}\left({\text{PTV}}_{i}\right)\right]$$

where *V* is the target volume. Formula (2) depends on the prescribed doses and the volumes of the targets, and does not depend on the other variables of the radiotherapy plan. If a plan has only one steep dose gradient, formula (2) for the I‐DVH is simplified into a unit step function, and the I‐DVH of PTV_1_ is shown in Fig. 2. If a plan has two steep dose gradients and two target volumes, the I‐DVH of PTV_1_ is simplified into a second‐order step function. The I‐DVH of PTV_1_ and PTV_2_ are shown in Fig. 3.

### 2.3 Evaluation of the new HI’s performance

Eighty‐eight clinical cases were used to evaluate the performance of the new HI, with these including 13 neck and head cases, 35 thorax cases, and 40 abdomen cases. For each case, a pair of treatment plans (plans A and B) was designed using a Pinnacle^3^ treatment planning system (version 9.10; Philips Medical Systems, Milpitas, CA). The planning goal was to deliver a prescribed dose to at least 95% of the target volume, while at the same time keeping the dose to organs at risk as low as possible. For head and neck plans, the maximum dose would not exceed 107% of prescribed dose. The dose constraints to normal tissues in head and neck plans are listed in Table 1.

**Table 1:** Dose constraints for the critical structures used in head and neck plans.

OAR	Dose constraint
Spinal cord PRV	*D*_max_ < 40 Gy
Brain stem PRV	*D*_max_ < 54 Gy
Lens	*D*_max_ < 9 Gy
Optic nerves, Chiasm	*D*_max_ < 54 Gy
Parotid	*V*_30_ < 50%
Temporal lobes	*D*_max_ < 54 Gy
TMJ	*D*_max_ < 50 Gy
Mandible	*D*_max_ < 60 Gy
Pituitary	*D*_max_ < 54 Gy
larynx	*D*_max_ < 40 Gy
Trachea	*D*_max_ < 40 Gy
Thyroid	*V*_40_ < 50%

Abbreviation: OAR = Organ at risk.

The homogeneity of the pair of plans was first evaluated by three physicists, with their judgments being considered as the evaluation standard for this study. In order to ensure that physicists were not disturbed by other dosimetric parameters when evaluating the dose homogeneity of the radiotherapy plans, only the DVH curves of the targets were afforded to them. The physicists were required to make one of three judgments: plan A was superior to plan B, plan A was inferior to plan B, or plan A was equivalent to plan B. If the three physicists gave three different opinions, the case was excluded. If the three physicists' evaluations were consistent or two of three physicists' evaluations were consistent, then the consistent evaluation result was used as the evaluation criteria of this study. The new HI and the other indices of *D*_max_/*D_p_*, *D*_5_/*D*_95_, (*D*_2_ − *D*_98_)/*D_p_*, (*D*_2_ − *D*_98_)/*D*_50_ and S‐index were also used to evaluate the homogeneity of the pair of radiotherapy plans. If the evaluation by the HI agreed with the judgments of the physicists, it was considered that the HI evaluation was accurate for that case, and if it was otherwise, it was considered inaccurate. The ratio of the number of accurate evaluations to the total number of evaluations was used to define the percentage accuracy. From the total of 88 cases, 4 cases were discarded, leaving 84 cases to be evaluated.

### 2.4 Statistical analysis

To determine whether there is a significant difference among the percentage accuracies of evaluation for the new HI, *D*_max_/*D_p_*, *D*_5_/*D*_95_, (*D*_2_ − *D*_98_)/*D*_p_, (*D*_2_ − *D*_98_)/*D*_50_, and S‐index, a Chi square test was performed for statistical analysis. The threshold for statistical significance was set at *P* < 0.05 (two‐tailed). All statistical analyses were performed using SPSS Version 19.0 (SPSS Inc., Chicago, IL).

## 3 RESULTS

### 3.1 Example cases

**Figure 4:**
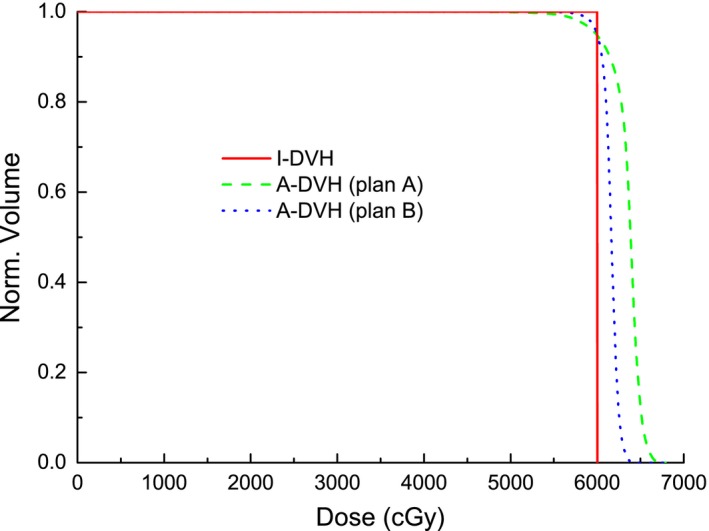
The I‐DVH (solid line), A‐DVHs of plans A (dash line) and B (dotted line) with one steep dose gradient under the same prescription. A‐DVH, achieved dose‐volume histogram curve; I‐DVH, ideal dose‐volume histogram.

Figure 4 shows an example of the new HI applied to two radiotherapy plans from the same case, plans with a single steep dose gradient. The prescribed dose for this case was 60 Gy delivered in 30 fractions. As can be seen in the figure, the two DVHs of plans A and B under the same prescription were significantly different from each other. Using the new HI, the homogeneity values of plans A and B were 0.94 and 0.97 respectively. The new HI clearly indicates a difference between plan A and B.

**Figure 5:**
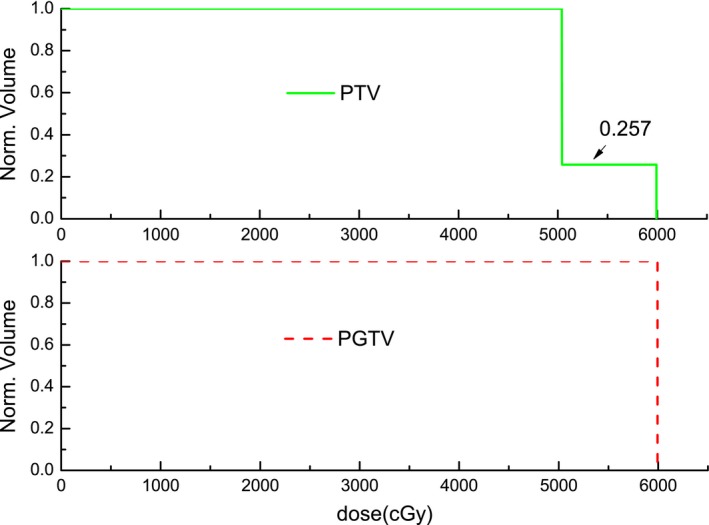
The I‐DVHs of PTV (solid line) and PGTV (dash line) for a radiotherapy plan with two steep dose gradients. I‐DVH, ideal dose‐volume histogram; PTV, planning target volume; PGTV, planning gross target volume.

**Figure 6:**
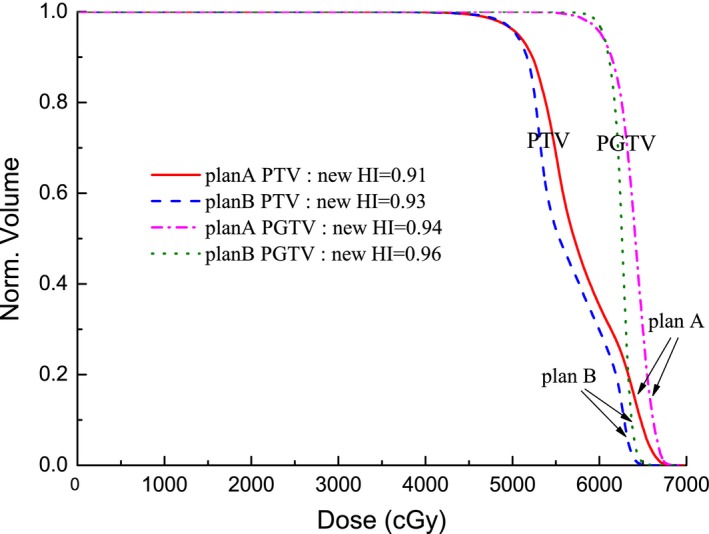
The A‐DVHs of plans A (solid line) and B (dash line) for PTV; A‐DVHs of plan A (dash dot line) and plan B (dot line) for PGTV. A‐DVHs, achieved dose‐volume histogram curve; PTV, planning target volume; PGTV, planning gross target volume.

Another example case is provided by a radiotherapy plan with two steep dose gradients for an esophageal cancer patient. The prescribed dose was 50.40 Gy delivered to the planning target volume (PTV) and 59.92 Gy simultaneously delivered to the planning gross target volume (PGTV), with the delivery being given in 30 fractions. The volume of the PTV was 810.26 cm^3^, and the volume of the intersection between the PTV and PGTV was 208.57 cm^3^. The I‐DVH of the PTV and PGTV are shown in Fig. 5. When the prescribed dose was larger than 50.40 Gy and less than 59.92 Gy, the value of I‐DVH of the PTV was 0.257, which was obtained from V(PGTV⊗PTV)/V(PTV). The A‐DVHs of plans A and B are shown in Fig. 6. Using the new HI definition, the homogeneity values of the PTV for plans A and B were 0.91 and 0.93 respectively. For the PGTV of plans A and B, the new HI values were 0.94 and 0.96, respectively. This result demonstrates that the new HI can accurately evaluate the dose homogeneity of a radiotherapy plan with two steep dose gradients.

### 3.2 Percentage accuracy of the evaluations

**Figure 7:**
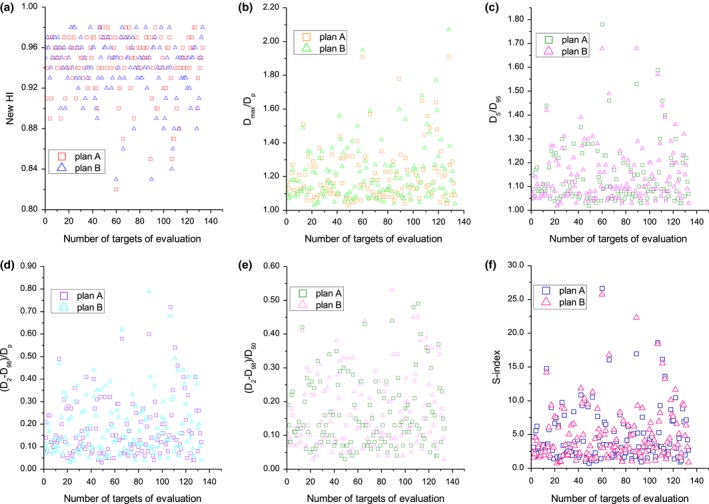
Scattered distributions of the values of the (a) new HI, (b) *D*_max_/*D_p_*, (c) *D*_5_/*D*_95_, (d) (*D*_2_ − *D*_98_)/*D_p_*, (e) (*D*_2_ − *D*_98_)/*D*_50_ and (f) S‐index of the plans A (square) and B (triangle). HI, homogeneity index.

Figure 7 shows the scattered distributions of the values of the new HI, *D*_max_/*D_p_*, *D*_5_/*D*_95_, (*D*_2_ − *D*_98_)/*D_p_*, (*D*_2_ − *D*_98_)/*D*_50_, and S‐index for all cases. As two plans were designed for each of the 84 cases, a total of 168 radiotherapy plans were evaluated by the physicists and HIs. Of the total of 84 cases, 52 used a radiotherapy plan with a single steep dose gradient, 21 used a plan with two steep dose gradients, 9 used a plan with three steep dose gradients, and 2 used a plan with four steep dose gradients; therefore a total of 258 values of HI were scattered in each small figure of Fig. 7. The values for the new HI, *D*_max_/*D_p_*, *D*_5_/*D*_95_, (*D*_2_ − *D*_98_)/*D_p_*, (*D*_2_ − *D*_98_)/*D*_50_, and S‐index ranged from 0.78–0.98, 1.03–2.07, 1.02–1.78, 0.03–0.79, 0.03–0.53, and 0.74–22.30, respectively (Fig. 7). Examination of the data in this Fig. 7 confirms that the homogeneity of target coverage is generally good, with most plans evaluated here having new HIs close to 1.0, having *D*_max_/*D_p_* and *D*_5_/*D*_95_ close to 1.0, and having (*D*_2_ − *D*_98_)/*D_p_* and (*D*_2_ − *D*_98_)/*D*_50_ close to 0.0. These results can verify the correctness of the calculation with each other.

**Figure 8:**
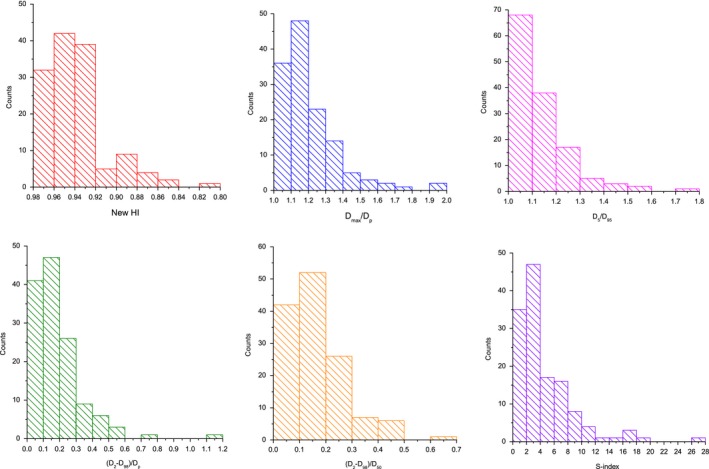
Statistical results of new HI, *D*_max_/*D_p_*, *D*_5_/*D*_95_, (*D*_2_ − *D*_98_)/*D_p_*, (*D*_2_ − *D*_98_)/*D*_50_, and S‐index for clinical plans. HI, homogeneity index.

The minimum, maximum, mean, median, and mode values, and percentage accuracy of the new HI, *D*_max_/*D_p_*, *D*_5_/*D*_95_, (*D*_2_ − *D*_98_)/*D_p_*, (*D*_2_ − *D*_98_)/*D*_50_, and S‐index are listed in Table 2. The lowest evaluation percentage accuracy was found with *D*_max_/*D_p_*, and the highest with the new HI. The newly defined HI had the highest accuracy of all the HIs, and this difference was statistically significant (*P* < 0.05). We made a statistical analysis for the values of the new HI, *D*_max_/*D_p_*, *D*_5_/*D*_95_, (*D*_2_ − *D*_98_)/*D_p_*, (*D*_2_ − *D*_98_)/*D*_50_, and S‐index for the clinical plans. The results are shown in Fig. 8, and data show skewed distribution for all HIs. According to the statistical results, distribution interval of 95% counts were selected as the recommended values of new HI, *D*_max_/*D_p_*, *D*_5_/*D*_95_, (*D*_2_ − *D*_98_)/*D_p_*, (*D*_2_ − *D*_98_)/*D*_50_, and S‐index for clinical plans, and they are listed in Table 3.

**Table 2:** The values of minimum, maximum, mean, median values, mode, and percentage accuracy of new HI, *D*_max_/*D_p_*, *D*_5_/*D*_95_, (*D*_2_ − *D*_98_)/*D_p_*, (*D*_2_ − *D*_98_)/*D*_50_, and S‐index.

Definition	Minimum value	Maximum value	Mean value	Median value	Mode	Percentage accuracy
New HI	0.78	0.98	0.94 ± 0.002	0.95	0.95	98.51%
*D*_max_/*D_p_*	1.03	2.07	1.21 ± 0.010	1.15	1.13	88.80%
*D*_5_/*D*_95_	1.02	1.78	1.14 ± 0.007	1.10	1.05	94.78%
(*D*_2_ − *D*_98_)/*D_p_*	0.03	0.79	0.19 ± 0.010	0.14	0.07	94.78%
(*D*_2_ − *D*_98_)/*D*_50_	0.03	0.53	0.17 ± 0.007	0.13	0.07	96.27%
S‐index	0.74	22.30	4.79 ± 0.251	3.27	1.39	97.01%
*P* value						0.007

Abbreviation: HI, homogeneity index.

**Table 3:** The recommended values of new HI, *D*_max_/*D_p_*, *D*_5_/*D*_95_, (*D*_2_ − *D*_98_)/*D_p_*, (*D*_2_ − *D*_98_)/*D*_50_, and S‐index for clinical plans.

Definition	New HI	*D*_max_/*D_p_*	*D*_5_/*D*_95_	(*D*_2_ − *D*_98_)/*D_p_*	(*D*_2_ − *D*_98_)/*D*_50_	S‐index
Recommended value	0.88–1.00	1.00–1.50	1.00–1.40	0.00–0.50	0.00–0.40	0.00–12.0

Abbreviation: HI, homogeneity index.

## 4 DISCUSSION

In this study, we defined a new HI and evaluated it alongside previously used HIs. The results show that the performance of the new HI was superior to the other five examined HIs of *D*_max_/*D_p_*, *D*_5_/*D*_95_, (*D*_2_ − *D*_98_)/*D_p_*, (*D*_2_ − *D*_98_)/*D*_50_, and S‐index. Accepting the evaluation of the three physicists as the gold standard, the new HI showed a high consistency with their evaluations, while reducing the percentage of inaccurate evaluations compared with the other examined HIs. Theoretically, the deficiencies in dose information with the conventional HIs would have negative implications^7^, ^12^, ^13^, as these conventional indices are based on a few points on the A‐DVH and could not afford the complete information of the whole dose distribution, although the exact clinical effects would be uncertain.

For the S‐index, which takes the whole DVH into consideration, even if only through the dispersion of the average dose of the target volume, the deviation of the ideal DVH is still unknown. However, by including IA, the new HI indicates an improved ability to evaluate the homogeneity of the radiotherapy plans by embodying information on the I‐DVH. More specifically, the new HI provides two kinds of information. First, it contains information on the ideal DVH, and second, it contains information on the achieved DVH. Therefore, oncologists and physicists could scale up the evaluation by including the new HI, thereby provide a potential improvement in the accurate evaluation of radiotherapy plans.

The impact of an increase in the number of prescribed doses in the radiotherapy plan on the dose homogeneity evaluated by HIs has not been addressed in previous literature. The new HI would be likely to provide more accurate evaluations compared with the previously described HIs because as the prescribed dose increases, the evaluations of the homogeneity would be more complex, while the new HI could avoid the problem as the IA is included as a parameter within it.

It should be noted that the percentage accuracy results are based on the interpretations of the physicists. We could not predict the accuracy of a physicist's assessment, so we therefore used three physicists to evaluate the homogeneity of each radiotherapy plan. The evaluations of three physicists are likely to be more accurate than those of a single physicist, and we eliminated those cases where the three physicists disagreed with each other. Although in this paper, the evaluations of three physicists are used as criteria to evaluate the accuracy of other methods for calculating the HI, we still cannot use them to evaluate the plans instead of using the proposed new and other methods to determine HI in clinical work. First, if the homogeneity of each plan is evaluated by three physicists, it will greatly increase the cost of manpower. Secondly, the physicist's judgment is only a qualitative evaluation, but it cannot give a specific quantitative evaluation.

Despite the benefits described above, the new HI also has limitation, and its evaluation sensitivity is not as high as the S‐index. This may affect the percentage accuracy of the evaluation. If two achieved DVHs are a little different, the quantitative evaluations achieved using the new HI could be the same. Therefore, further work to improve the sensitivity of the evaluation is necessary.

## 5 CONCLUSIONS

In the present study, a new HI definition is given, one which evaluates the dose homogeneity through the ratio of the square of the OA to the product of the IA and AA. The applicability of the new HI is shown by using it in two clinical examples. Homogeneity evaluations in 84 cases using the new HI, and *D*_max_/*D_p_*, *D*_5_/*D*_95_, (*D*_2_ − *D*_98_)/*D_p_*, (*D*_2_ − *D*_98_)/*D*_50_, and S‐index, with the evaluation of three physicists as the gold standard, showed that the new HI had the most accurate evaluation performance, with a percentage accuracy reaching 98.51%, a value higher than achieved with the other HIs evaluated in this study. This result was statistically significant (*P* < 0.05) according to Pearson's chi‐squared test.

## CONFLICT OF INTEREST

None.

## References

[1] Webb S , Nahum AE . A model for calculating tumour control probability in radiotherapy including the effects of inhomogeneous distributions of dose and clonogenic cell density. Phys Med Biol. 1993;20:653–666. 10.1088/0031-9155/38/6/001 8346278

[2] Jackson A , Kutcher GJ , Yorke ED . Probability of radiation‐induced complications for normal tissues with parallel architecture subject to nonuniform irradiation. Med Phys. 1993;20:613–625. 8350812 10.1118/1.597056

[3] Goitein Michael . Causes and consequences of inhomogeneous dose distributions in radiation therapy. Int J Radiation Oncol Biol Phys. 1986;12:701–704. 10.1016/0360-3016(86)90084-2 3700176

[4] Kestin LL , Sharpe MB , Frazier RC , et al. Intensity modulation to improve dose uniformity with tangential breast radiotherapy: initial clinical experience. Int J Radiation Oncol Biol Phys. 2000;5:1559–1568. 10.1016/s0360-3016(00)01396-1 11121662

[5] Vaarkamp J , Krasin M . Reduction of target dose inhomogeneity in IMRT treatment planning using biological objective functions. Int J Radiat Biol Phys. 2001;49:1518–1520. 10.1016/s0360-3016(00)01538-8 11293435

[6] Kataria T , Sharma K , Subramani V , Karrthick KP , Bisht SS . Homogeneity Index: an objective tool for assessment of conformal radiation treatments. J Med Phys. 2012;37:207–213. 23293452 10.4103/0971-6203.103606 PMC3532749

[7] Lee S , Cao YJ , Kim CY .Physical and radiobiological evaluation of radiotherapy treatment plan, evolution of ionizing radiation research. Dr. Mitsuru N (Ed.), Croatia, InTech; 2015, DOI: 10.5772/60846.

[8] Tol JP , Delaney AR , Dahele M , Slotman BJ , Verbakel WFAR . Evaluation of a knowledge‐based planning solution for head and neck cancer. Int J Radiation Oncol Biol Phys. 2015;91:612–620. 10.1016/j.ijrobp.2014.11.014 25680603

[9] Shaw E , Kline R , Gillin M , et al. Radiation therapy oncology group: radiosurgery quality assurance guidelines. Int J Radiat Oncol Biol Phys. 1993;27:1231–1239. 8262852 10.1016/0360-3016(93)90548-a

[10] Weiss E , Siebers JV , Keall PJ . An analysis of 6‐MV versus 18‐MV photon energy plans for intensity‐modulated radiation therapy (IMRT) of lung cancer. Radiother Oncol. 2007;82:55–62. 17150271 10.1016/j.radonc.2006.10.021

[11] Wu Q , Mohan R , Morris M , Lauve A , Schmidt‐Ullrich R . Simultaneous integrated boost intensity‐modulated radiotherapy for locally advanced head‐and‐neck squamous cell carcinomas. I: dosimetric results. Int J Radiat Oncol Biol Phys. 2003;56:573–585. 12738335 10.1016/s0360-3016(02)04617-5

[12] Commission on Radiation Units and Measurements . Measurements. Report 83. Prescribing, recording, and reporting photon‐beam intensity‐modulated radiation therapy (IMRT). Oxford University Press; 2010.

[13] Myonggeun Y , Sung YP , Dongho S , et al. A new homogeneity index based on statistical analysis of the dose–volume histogram. J Appl Clin Med Phys. 2007;8:9–17. 17592460 10.1120/jacmp.v8i2.2390 PMC5722417

[14] Antonella B , Giorgio A . delle Canne S , et al. Comparison between the ideal reference dose level and the actual reference dose level from clinical 3D radiotherapy treatment plans. Radiother Oncol. 2009;92:68–75. 19328571 10.1016/j.radonc.2009.02.018

